# Regional disparities in postnatal care among mothers aged 15-49 years old: An analysis of the Indonesian Demographic and Health Survey 2017

**DOI:** 10.12688/f1000research.50938.2

**Published:** 2021-08-16

**Authors:** Mochammad Nur Cahyono, Ferry Efendi, Harmayetty Harmayetty, Qorinah Estiningtyas Sakilah Adnani, Hsiao Ying Hung

**Affiliations:** 1 Faculty of Nursing, Universitas Airlangga, Surabaya, Indonesia; 2 Department of Midwifery, Karya Husada Institute of Health Science, Kediri, Indonesia; 3 Quality Maternal & Newborn Care Research Alliance, Yale University, Connecticut, USA; 4 Department of Nursing, College of Medicine, National Cheng Kung University, Tainan, Taiwan

**Keywords:** postnatal care, regional disparities, reduced inequalities

## Abstract

**Background:** In Indonesia, maternal mortality remains high, significantly 61.59% occur in the postnatal period. Postnatal care (PNC) provision is a critical intervention between six hours and 42 days after childbirth and is the primary strategy to reduce maternal mortality rates. However, underutilisation of PNC in Indonesia still remains high, and limited studies have shown the regional disparities of PNC in Indonesia.

**Methods:** This study aims to explore the gaps between regions in PNC service for mothers who have had live births during the last five years in Indonesia. This study was a secondary data analysis study using the Indonesian Demographic and Health Survey (IDHS) in 2017. A total of 13,901 mothers aged 15-49 years having had live births within five years were included. Chi-squared test and binary logistic regression were performed to determine regional disparities in PNC.

**Results:** Results indicated that the prevalence of PNC service utilisation among mothers aged 15-49 years was 70.94%. However, regional gaps in the utilisation of PNC service were indicated. Mothers in the Central of Indonesia have used PNC services 2.54 times compared to mothers in the Eastern of Indonesia (OR = 2.54; 95% CI = 1.77-3.65, p<0.001). Apart from the region, other variables have a positive relationship with PNC service, including wealth quintile, accessibility health facilities, age of children, childbirth order, mother's education, maternal occupation, spouse's age, and spouse's education.

**Conclusion:** The results suggest the need for national policy focuses on service equality, accessible, and reliable implementation to improve postnatal care utilisation among mothers to achieve the maximum results for the Indonesian Universal Health Coverage plan.

## Introduction

Maternal morbidity and mortality are serious global health challenges. In 2019, the World Health Organization (WHO) revealed that 94% of maternal mortality occurred in low and middle-income countries, of which Indonesia is one ( World Health Organization, 2019; World Health Organization *et al.*, 2015). During 2000 and 2017, the maternal mortality ratio plunged by about 38% worldwide. Even with this decline, Sub-Saharan Africa and Southern Asia accounted for approximately 86% of maternal deaths worldwide. Southern Asia alone accounted for nearly one-fifth (58,000), which demonstrated the struggle to improve maternal health ( World Health Organization, 2019; World Health Organization *et al.*, 2015). In the Indonesia context, the government determined a target to reach an important goal of reducing maternal mortality rate to 102 per 100,000 live births in 2015 ( Bappenas, 2015; Ministry of Health Republic of Indonesia, 2018). Despite the significant efforts to expand maternal health programmes, recent evidence showed that Indonesia was off-track to reach the Millenium Development Goals (MDGs) target by 2015. In 2015, maternal mortality deaths in Indonesia were three times higher than the MDGs target ( Ministry of Health Republic of Indonesia, 2018; UNICEF & World Health Organization, 2015; World Health Organization *et al.*, 2015). Hence, reducing maternal mortality ratio to less than 70 per 100,000 by 2030 as one of the Sustainable Development Goals (SDGs) targets could be a critical challenge for Indonesia ( UNICEF & World Health Organization, 2015; United Nations, 2017; World Health Organization, 2012).

Most maternal mortality deaths are preventable or treatable if skilled healthcare, such as midwives, is provided during the postnatal period. Critical interventions during the postnatal period must be delivered to prevent maternal mortality deaths ( Lawn *et al.*, 2016; World Health Organization *et al.*, 2015). In the Indonesian setting, the cause of maternal mortality is predominantly due to postpartum haemorrhage, followed by indirect causes, such as heart disease, severe anaemia, malaria, HIV/ AIDS and hepatitis. Many factors have been linked to interventions of postpartum haemorrhage which cause emergency cases, and skilled health care required to respond effectively to emergencies ( Adisasmita *et al.*, 2015; Mahmood *et al.*, 2018). However, PNC remains a critical intervention to reduce maternal deaths in Indonesia. A new health programme called EMAS or Expanding Maternal and Neonatal Survival) was implemented by the Indonesian government in 2012, focused on improving maternal health care. The programme aimed at ensuring that every woman has access to quality maternal healthcare, including childbirth assistance by skilled health personnel in healthcare facilities (a target of 2018 strategic plan: 82%), four visits of Antenatal care (ANC) (78% ), PNC, and providing ANC (87%). However, the national data revealed that utilisation of PNC among 34 provinces in Indonesia remain varied and was considered lower compared to the childbirth assistance by skilled health personnel coverage. Socioeconomic, geographical, and demographic factors influence the underutilisation of PNC. Current systematic reviews show that levels of education, poverty, and limitations of access to PNC services are common issues in low-and middle-income countries linked to inequities in the use of PNC services ( Langlois *et al.*, 2015).

The national data of Indonesia in 2018 revealed that the average percentage of PNC visits for the first time in Indonesia was 93.3%. The highest percentage of visits occurred in Yogyakarta (99.6%), and the lowest percentage of visits was in Papua (56.3%). However, there were regional gaps in PNC visits across the provinces in Indonesia. Also, PNC utilisation in rural areas was lower than in urban areas of Indonesia ( Kementerian Kesehatan Republik Indonesia, 2018; Ministry of Health Republic of Indonesia, 2018; Probandari *et al.*, 2017). It is worth noting that 61.59 % of maternal mortality rates occurred during the postnatal period in Indonesia. Additionally, evidence shows that the quality of PNC is lower in most districts and cities among the Eastern Region in Indonesia. PNC must be performed a minimum of three times: within the first six hours to the third day after the delivery, from the fourth day to the 28th after the delivery and the 29th day to the 42nd day after childbirth. The standards of PNC including examination for vital signs; the apex of the uterus; lochia and other per vagina fluids; breasts and counselling for exclusive breastfeeding; provision of communication, information about and education of PNC, and family planning ( Ministry of Health Republic of Indonesia, 2018; Probandari *et al.*, 2017).

Several studies show that regional disparities in PNC occur in several countries. In Ethiopia, there were differences in each region and variations at regional levels at the utilisation of PNC among women ( Sisay *et al.*, 2019). PNC service in Zambia was also reported to experience regional disparities ( Jacobs *et al.*, 2017). In the same vein, in Bangladesh, disparities in the utilisation of maternal health services were also reported ( Raheem *et al.*, 2019). However, research focused on regional disparities on PNC among mothers aged 15–49 years old in Indonesia are not well investigated. This study was conducted to analyse the gap between regions in PNC service utilisation among mothers aged 15 – 49 years old who have had live births during the last five years in Indonesia. This study is significant because it can be a source of information and a reference regarding regional disparities in the utilisation of PNC services in Indonesia. This research could complete a bigger picture for consideration in resolving discrepancies in maternal services in Indonesia.

## Methods

This study was a secondary data analysis using the most recent data from the 2017 Indonesian Demographic Data Survey (IDHS). In this study, unit analysis data consist of women aged 15–49 years old having had live births in the last five years preceding the survey.

The purpose of the cross-sectional study of the IDHS conducted by the Inner City Fund (ICF) international together with the national implementer in Indonesia was to offer up-to-date projections of fundamental demographic and health indicators. The IDHS study demonstrates a broad overview of population problems in Indonesia.

We utilised the data conducted by the national and provincial representatives. This cross-sectional study represents 1,970 census blocks in urban and rural areas of Indonesia. The census block obtained 59,100 female respondents aged 15–49 years old. The survey employed a two-stage stratified cluster sampling method. The first stage was the selection of several census blocks by systematic sampling proportional size. In the second stage, 25 ordinary households were selected with systematic sampling from the listing. In this study, a sample of 13,901 women aged 15–49 years from 34 provinces in Indonesia was analysed. The inclusion criteria were taken from IDHS that included all women aged 15–49 years who had given birth in the last five years. The exclusion criteria were whether the variables incomplete or missing. In order to allow replicate the DHS data, the guide for using datasets for DHS analysis is available at https://dhsprogram.com/data/Using-DataSets-for-Analysis.cfm.

### Ethical consideration

Ethical review boards approved ethical clearance for the Inner City Fund OCR Macro (number 45 CFR 46) and the national board review from the Ministry of Health of Republic Indonesia. Before the survey, an informed consent was obtained from the respondents based on voluntary participation.

### Variables

The dependent variable of this study was PNC visits. According to the recommendation of the Ministry of Health Republic of Indonesia, PNC must be performed at minimum three times: at the first six hours to the third day after the delivery, on the fourth day to the 28th after the delivery and the 29th day to the 42nd day after childbirth ( Ministry of Health Republic of Indonesia, 2018). This data was based on the mother’s perception of PNC utilisation during the postnatal period. Independent variables analysed in this study were the related geographic and socioeconomic factors, including a region of residence, the place of residence, wealth quintile, health insurance, access to a health facility, age, gender, birth rank, education, and occupation.

The residence region was grouped as six regions, namely Sumatera, Java, Bali & Nusa Tenggara, Kalimantan, Sulawesi, Maluku & Papua, and of which were also categorised as Western Indonesia, Central Indonesia and Eastern Indonesia. The place of residence was determined as rural and urban areas. The wealth quintile of households was set into five categories: poorest, poorer, middle, richer, and richest. The wealth quintile of households was scored based on wealth criteria ( DHSProgram, 2016). Health insurance was divided into two categories, namely yes and no. Access to the health facility was categorised into two, namely difficult and not. Children’s age was divided into five categories: less than one month, one month, two months, three months, four months. The gender of the child, namely female and male. Birth rank was categorised as a first child, second, third, fourth and so on. Mother’s age was divided into six categories: 15–24, 25–29, 30–34, 35–39, 40–49, and 45–49, while spouse’s age was divided into seven categories: 11–20, 21–30, 31–40, 41–50, 51–60, 61–70, 71–80. Mother’s and spouses' education levels were grouped into no education, primary, secondary, and higher education. Mother’s and spouse’s occupation was divided into two categories: not working and working. Determination of each category on the variables based on DHS report ( BKKBN-BPS-Kemenkes-ICF, 2018) which was adjusted to the minimum number of sample on each category to meet the statistical assumption. 

### Statistical analysis

Data were analysed using STATA version 16.0 by conducting descriptive analysis. The chi-square test was performed to determine variables correlated to the PNC utilisation. Binary logistic regression was utilised to determine disparity in this study. Measurement of associations among variables was expressed as Odds Ratio (OR) and 95% Confidence Interval (CI). Significant variables were tested with a p-value of 0.05 and 95% CI, which are considered the disparity in PNC among mothers aged 15–49 years in Indonesia.

## Results

A total of 13,901 women aged 15–49 years old with live births in the last five years preceding the survey were interviewed. Table 1 shows the bivariate analysis that there were ten categories among some variables associated with the utilisation of PNC visits (p-value < 0.05). These variables include geographic factors, region, socio-economy (wealth quintile and access to the health facility), children (age of child and birth rank), mother factors (age, education and occupation), spouse factors (age, education, and occupation). Residence, socioeconomic (health insurance ownership), child gender, mother’s age and spouse’s occupation did not show associations with the utilisation of PNC visits among mothers aged 15–49 years old in Indonesia ( Table 1). More detail results can be found in Table 1.

**Table 1.** Socio-demographic characteristic of participants by utilization of PNC in Indonesia (n=13,901).

Characteristic	Utilization of PNC				X ^2^	*p-value*
	No		Yes			
	n	%	n	%		
**The geographic factors**						
**Indonesia**						
Western Indonesia	3265	29.1	7946	70.9	41.45 ***	0.00
Central Indonesia	611	26.5	1693	73.5		
Eastern Indonesia	164	42.4	222	57.6		
**Region**						
Sumatera	1056	34.3	2027	65.7	116.36 ***	0.00
Java	2028	26.3	5691	73.7		
Bali & Nusa Tenggara	232	26.5	642	73.5		
Kalimantan	291	33.5	578	66.5		
Sulawesi	270	27.8	701	72.2		
Maluku & Papua	164	42.4	222	57.6		
**Place of residence**						
Rural	2051	28.6	5116	71.4	1.45	0.49
Urban	1989	29.5	4745	70.5		
**Socioeconomic factors**						
**Wealth quintile**						
Poorest	928	34.6	1757	65.4	58.56 ***	0.00
Poorer	827	29.5	1980	70.5		
Middle	781	26.9	2127	73.1		
Richer	753	26.3	2109	73.7		
Richest	751	28.4	1888	71.6		
**Health insurance**						
No	1713	29.8	4026	70.2	3.00	0.19
Yes	2326	28.5	5835	71.5		
**Access to the health** **facility**						
Difficult	555	36.0	985	64.0	41.46 ***	0.00
Not	3485	28.2	8877	71.8		
**Child’s factors**						
**Age of child (in month)**						
Less than one month	1007	33.0	2045	67.0	58.13 ***	0.00
One month	938	29.9	2198	70.1		
Two months	840	30.0	1956	70.0		
Three months	631	24.7	1929	75.3		
Four months	623	26.4	1733	73.6		
**Sex of child**						
Male	2118	29.9	4963	70.1	5.11	0.06
Female	1922	28.2	4898	71.8		
**Birth rank**						
First child	1251	27.1	3368	72.9	41.25 ***	0.00
Second	1400	28.7	3469	71.3		
Third	761	29.0	1864	71.0		
Fourth and more	628	35.1	1160	64.9		
**Mother’s factors**						
**Age of mother in year**						
15–24	796	30.6	1808	69.4	6.26	0.52
25–29	1032	29.2	2502	70.8		
30–34	995	27.7	2594	72.3		
35–39	808	29.0	1975	71.0		
40–44	336	29.3	809	70.7		
45–49	73	29.5	174	70.5		
**Mother’s level of education**						
No education	63	50.0	63	50.0	55.19 ***	0.00
Primary	1117	31.2	2458	68.8		
Secondary	2357	28.9	5803	71.1		
Higher	503	24.6	1537	75.4		
**Mother’s occupation**						
Working	1917	27.2	5136	72.8	24.97 ***	0.0001
Not working	2123	31.0	4725	69.0		
**Spouse’s factor**						
**Age of spouse in year**						
11–20	44	45.8	52	54.2	23.83 **	0.0068
21–30	1164	29.6	2772	70.4		
31–40	1901	28.2	4843	71.8		
41–50	817	29.2	1985	70.8		
51–60	106	35.7	192	64.3		
61–70	8	33.8	15	66.2		
71–80	0	0.0	2	100.0		
**Spouse’s level of** **education**						
No education	51	37.0	86	63.0	39.21 ***	0.0001
Primary	1254	32.5	2610	67.5		
Secondary	2255	28.0	5803	72.0		
Higher	479	26.0	1362	74.0		
**Spouse’s occupation**						
Working	4016	29.1	9799	70.9	0.15	0.72
Not working	23	27.2	63	72.8		

*p<0.05; **p<0.01;***p<0.001

Table 2 shows that the least distribution of respondents is in the Eastern of Indonesia. Nearly half of participants who live in the Eastern of Indonesia did not use the PNC services. More than half of participants who live in the Eastern of Indonesia was classified as the lowest. Interestingly, most participants who live in the Eastern of Indonesia stated that there was no problem with access to the health facility. More detail characteristics of participants can be found in Table 2.

**Table 2.** Socio-demographic characteristic of study participants PNC in Indonesia based on region (n=13,901).

	Indonesia							
The use of PNC	Western Indonesia		Central Indonesia		Eastern Indonesia		Total	
	n	%	N	%	n	%	n	%
No	3265	29.12	611	26.51	164	42.45	4040	29.06
Yes	7946	70.88	1693	73.49	222	57.55	9861	70.94
Wealth quintile								
Poorest	1686	15.04	772	33.51	227	58.75	2685	19.32
Poorer	2224	19.84	516	22.40	67	17.30	2807	20.19
Middle	2465	21.99	403	17.48	40	10.42	2908	20.92
Richer	2516	22.44	314	13.63	32	8.28	2862	20.59
Richest	2320	20.69	299	12.98	20	5.25	2639	18.99
Health insurance								
No	4731	42.20	870	37.77	138	35.77	5739	41.29
Yes	6480	57.80	1434	62.23	248	64.23	8162	58.71
Access to the health facility								
Difficult	1176	10.49	294	12.75	70	18.03	1539	11.07
Not	10035	89.51	2010	87.25	317	81.97	12361	88.93
Age of child (in month)								
Less than one month	2461	21.95	495	21.48	97	25.02	3052	21.96
One month	2533	22.59	509	22.11	93	24.21	3136	22.56
Two months	2230	19.89	485	21.07	82	21.17	2797	20.12
Three months	2084	18.59	411	17.84	64	16.64	2560	18.42
Four months	1903	16.98	403	17.50	50	12.96	2356	16.95
Sex of child								
Male	5671	50.58	1208	52.42	203	52.56	7081	50.94
Female	5540	49.42	1096	47.58	183	47.44	6819	49.06
Birth rank								
First child	3843	34.28	686	29.77	90	23.28	4619	33.23
Second	4015	35.81	752	32.65	101	26.27	4869	35.03
Third	2100	18.74	453	19.68	71	18.45	2625	18.88
Fourth and more	1252	11.17	412	17.89	124	32.00	1788	12.86
Age of mother in year								
15–24	2080	18.55	445	19.33	79	20.46	2604	18.73
25–29	2853	25.44	579	25.14	102	26.28	3533	25.42
30–34	2916	26.01	577	25.05	95	24.69	3589	25.82
35–39	2277	20.31	435	18.87	72	18.54	2783	20.02
40–44	902	8.04	214	9.29	29	7.45	1145	8.23
45–49	183	1.63	53	2.32	10	2.57	247	1.77
Mother’s level of education								
No education	63	0.56	43	1.87	21	5.35	127	0.91
Primary	2855	25.47	633	27.47	87	22.52	3575	25.72
Secondary	6739	60.11	1213	52.66	208	53.84	8160	58.70
Higher	1554	13.86	415	18.00	71	18.29	2039	14.67
Mother’s occupation								
Working	5473	48.82	1352	58.68	228	59.12	7053	50.74
Not working	5738	51.18	952	41.32	158	40.88	6847	49.26
Age of spouse in year								
11–20	70	0.63	20	0.85	6	1.48	96	0.69
21–30	3135	27.96	672	29.15	129	33.46	3936	28.31
31–40	5474	48.83	1093	47.43	177	45.91	6744	48.52
41–50	2295	20.47	445	19.31	62	16.06	2802	20.16
51–60	220	1.96	67	2.91	11	2.92	298	2.14
61–70	15	0.14	7	0.31	1	0.17	23	0.17
71–80	1	0.01	1	0.03	0	0.00	2	0.01
Spouse's level of education								
No education	73	0.65	49	2.14	15	3.77	137	0.99
Primary	3066	27.35	716	31.10	81	20.98	3864	27.80
Secondary	6653	59.34	1175	51.01	230	59.65	8058	57.97
Higher	1418	12.65	363	15.75	60	15.60	1841	13.25
Spouse's occupation								
Working	11154	99.49	2285	99.20	376	97.32	13815	99.38
Not working	57	0.51	18	0.80	10	2.68	86	0.62
**Total**	**11211**	**100.00**	**2304**	**100.00**	**386**	**100.00**	**13901**	**100.00**

Table 3 reveals that nearly half of participants who live in Sumatera, Kalimantan, Maluku and Papua did not use the PNC service. The highest number of participants classified as lowest was found in Maluku & Papua. For health insurance ownership, more participants who live in Kalimantan did not have health insurance. The highest number of participants having four and more children per household were found in Maluku & Papua ( Table 3).

**Table 3.** Sociodemographic characteristic of participants PNC in Indonesia based on region (n=13,901).

	Region												
The use of PNC	Sumatera		Java		Bali & Nusa Tenggara		Kalimantan		Sulawesi		Maluku & Papua		Total
	n	%	n	%	n	%	n	%	n	%	n	%	n
No	1056	34.25	2028	26.27	232	26.52	291	33.46	270	27.79	164	42.45	4040
Yes	2027	65.75	5691	73.73	642	73.48	578	66.54	701	72.21	222	57.55	9861
Wealth quintile													
Poorest	711	23.05	838	10.85	357	40.83	211	24.34	342	35.21	227	58.75	2685
Poorer	725	23.52	1401	18.16	172	19.67	203	23.43	238	24.54	67	17.30	2807
Middle	657	21.32	1722	22.31	121	13.88	205	23.60	162	16.72	40	10.42	2908
Richer	546	17.72	1914	24.80	115	13.10	143	16.41	112	11.57	32	8.28	2862
Richest	444	14.39	1844	23.89	109	12.52	106	12.22	116	11.96	20	5.25	2639
Health insurance													
No	1259	40.83	3238	41.95	356	40.74	437	50.33	311	32.04	138	35.77	5739
Yes	1824	59.17	4481	58.05	518	59.26	431	49.67	659	67.96	248	64.23	8162
Access to the health facility													
Difficult	373	12.10	744	9.64	104	11.88	104	11.98	144	14.87	70	18.03	1539
Not	2710	87.90	6974	90.36	770	88.12	764	88.02	826	85.13	317	81.97	12361
Age of child (in month)													
Less than one month	731	23.70	1638	21.23	174	19.88	178	20.50	235	24.17	97	25.02	3052
One month	679	22.03	1770	22.93	193	22.05	175	20.18	225	23.22	93	24.21	3136
Two months	628	20.37	1515	19.62	184	21.01	201	23.15	188	19.34	82	21.17	2797
Three months	578	18.76	1438	18.63	165	18.91	152	17.54	162	16.65	64	16.64	2560
Four months	467	15.13	1358	17.59	159	18.15	162	18.63	161	16.62	50	12.96	2356
Sex of child													
Male	1589	51.55	3886	50.35	467	53.40	431	49.65	505	52.02	203	52.56	7081
Female	1494	48.45	3832	49.65	407	46.60	437	50.35	466	47.98	183	47.44	6819
Birth rank													
First child	882	28.60	2838	36.77	251	28.67	260	29.97	298	30.74	90	23.28	4619
Second	1025	33.24	2845	36.87	308	35.26	313	36.05	276	28.43	101	26.27	4869
Third	655	21.25	1357	17.58	160	18.26	178	20.44	205	21.11	71	18.45	2625
Fourth and more	521	16.91	678	8.79	156	17.81	118	13.54	191	19.72	124	32.00	1788
Age of mother in year													
15–24	512	16.60	1474	19.10	149	17.07	180	20.76	210	21.64	79	20.46	2604
25–29	783	25.41	1953	25.30	220	25.16	248	28.60	227	23.39	102	26.28	3533
30–34	886	28.73	1931	25.02	227	26.00	221	25.47	228	23.51	95	24.69	3589
35–39	604	19.59	1610	20.86	173	19.83	136	15.70	188	19.39	72	18.54	2783
40–44	249	8.09	625	8.09	84	9.57	62	7.12	96	9.91	29	7.45	1145
45–49	49	1.59	126	1.63	21	2.36	20	2.35	21	2.17	10	2.57	247
Mother's level of education													
No education	31	1.01	24	0.31	32	3.70	9	1.01	10	1.00	21	5.35	127
Primary	721	23.40	1988	25.76	250	28.58	269	30.98	260	26.75	87	22.52	3575
Secondary	1782	57.81	4739	61.40	445	50.94	479	55.17	506	52.14	208	53.84	8160
Higher	548	17.78	967	12.53	147	16.78	112	12.85	195	20.10	71	18.29	2039
Mother’s occupation													
Working	1684	54.63	3590	46.51	559	64.00	448	51.63	543	55.96	228	59.12	7053
Not working	1399	45.37	4129	53.49	315	36.00	420	48.37	427	44.04	158	40.88	6847
Age of spouse in year													
11–20	23	0.74	42	0.54	8	0.89	9	1.04	9	0.90	6	1.48	96
21–30	829	26.90	2169	28.10	244	27.87	258	29.67	307	31.61	129	33.46	3936
31–40	1557	50.50	3725	48.26	435	49.80	412	47.42	438	45.15	177	45.91	6744
41–50	606	19.66	1627	21.08	164	18.78	159	18.30	184	18.96	62	16.06	2802
51–60	63	2.04	146	1.89	22	2.53	30	3.42	26	2.72	11	2.92	298
61–70	4	0.12	11	0.14	1	0.13	1	0.15	6	0.57	1	0.17	23
71–80	1	0.04	0	0.00	0	0.00	0	0.00	1	0.08	0	0.00	2
Spouse's level of education													
No education	21	0.67	46	0.60	30	3.45	11	1.29	14	1.49	15	3.77	137
Primary	786	25.50	2140	27.72	266	30.38	267	30.74	324	33.43	81	20.98	3864
Secondary	1865	60.51	4564	59.14	430	49.20	490	56.43	478	49.23	230	59.65	8058
Higher	410	13.31	968	12.55	148	16.97	100	11.55	154	15.85	60	15.60	1841
Spouse’s occupation													
Working	3072	99.65	7673	99.41	865	98.93	865	99.55	964	99.33	376	97.32	13815
Not working	11	0.35	45	0.59	9	1.07	4	0.45	6	0.67	10	2.68	86
**Total**	**3083**	100.00	**7718**	100.00	**874**	100.00	**869**	100.00	**970**	100.00	**386**	100.00	**13901**

In multivariate analysis, the participants who live in the Central of Indonesia utilised PNC services 2.54 times more than the participants who live in the Western of Indonesia (OR = 2.54; 95% CI = 1.77-3.65). The participants who live in the Eastern of Indonesia had 0.71 fewer odds than participants in Indonesia’s Western (OR = 0.71; 95% CI = 0.52-0.96). The participants who live in Java were 1.46 times more likely to use PNC services (OR = 1.46; 95% CI = 1.26-1.69) compared to participants living in Sulawesi (OR = 0.53; 95% CI = 0.35-0.80). ( Table 4). Details of the results of multivariate analysis shown in Table 4.

**Table 4.** Binary logistic regression of PNC utilisation among mothers aged 15–49 years old in Indonesia based on geographical location.

Variable	The use of PNC			
	*P*	OR	95% CI	
			*lower*	*upper*
**Indonesia**				
Western Indonesia	*Ref.*	1.0		
Central Indonesia	0.00 ***	2.54	1.77	3.65
Eastern Indonesia	0.03 *	0.71	0.52	0.96
**Region**				
Sumatera	*Ref.*	1.0		
Java	0.00 ***	1.46	1.26	1.69
Bali & Nusa Tenggara	0.007 **	0.57	0.38	0.86
Kalimantan	0.007 **	0.66	0.48	0.89
Sulawesi	0.002 **	0.53	0.35	0.80
Maluku & Papua	*Omitted*			

* *p*<0.05; ** *p*<0.01; *** *p*<0.001.

Table 5 reveals the middle category women based on wealth index had PNC service’s utilization increased by 1.25 greater odds (OR = 1.25; 95% CI = 1.07-1.47) more than the richer category mothers (OR = 1.23; 95% CI = 1.04-1.45). Participants who thought that access to the health facility was not a problem had an odds ratio of 1.29 greater than participants who considered access to the health facility to be a major problem in using PNC service (OR = 1.29; 95% CI = 1.10-1.51). Mothers having children aged three months had used PNC service 1.43 times (OR = 1.43; 95% CI = 1.23-1.66) more than mothers with children aged four months (OR = 1.30; 95% CI = 1.12-1.51). Mothers who had a second child had utilized PNC service 0.88 times (OR = 0.88; 95% CI = 0.78-0.99) more than mothers who had a third child (OR = 0.86; 95% CI = 0.74-0.99). Mothers who had higher education had 2.11 times the chance to utilize PNC visits (OR = 2.11; 95% CI = 1.24-3.59) compared to those with lower-level education (OR = 1.80; 95% CI = 1.09-2.98). Mothers with spouse’s aged 41–50 had a higher chance of utilization PNC visits (OR = 2.18; 95% CI = 1.31-3.63) compared to mothers with spouse’s aged 31–40 (OR = 2.07; 95% CI = 1.26-3.40). Details of the results of a multivariate analysis shown in Table 5.

**Table 5.** Binary logistic regression of PNC utilisation among mothers aged 15–49 years old in Indonesia.

Variable	The use of PNC			
	*P*	OR	95% CI	
			*lower*	*upper*
**Wealth quintile**				
Poorest	*Ref.*	1.0		
Poorer	0.07	1.15	0.99	1.35
Middle	0.007 **	1.25	1.07	1.47
Richer	0.017 *	1.23	1.04	1.45
Richest	0.86	1.02	0.85	1.22
**Access to the health** **facility**				
Difficult	*Ref.*	1.0		
Not	0.001 **	1.29	1.10	1.51
**Age of child (in month)**				
Less than one month	*Ref.*	1.0		
One month	0.06	1.13	0.99	1.28
Two months	0.11	1.11	0.98	1.26
Three months	0.00 ***	1.43	1.23	1.66
Four months	0.001 **	1.30	1.12	1.51
**Birth rank**				
First child	*Ref.*	1.0		
Second	0.04 *	0.88	0.78	0.99
Third	0.04 *	0.86	0.74	0.99
Fourth and more	0.00 ***	0.68	0.58	0.81
**Mother’s level of** **education**				
No education	*Ref.*	1.0		
Primary	0.02 *	1.80	1.09	2.98
Secondary	0.03 *	1.77	1.06	2.96
Higher	0.006 **	2.11	1.24	3.59
**Mother’s occupation**				
Working	*Ref.*	1.0		
Not working	0.007 **	0.87	0.79	0.96
**Age of spouse in year**				
11–20	*Ref.*	1.0		
21–30	0.016 *	1.83	1.12	3.00
31–40	0.004 **	2.07	1.26	3.40
41–50	0.003 **	2.18	1.31	3.63
51–60	0.06	1.73	0.97	3.07
61–70	0.17	2.19	0.72	6.68
71–80	*Omitted*			
**Spouse’s level of** **education**				
No education	*Ref.*	1.0		
Primary	0.98	1.01	0.67	1.51
Secondary	0.50	1.15	0.76	1.74
Higher	0.48	1.17	0.76	1.83

* *p*<0.05; ** *p*<0.01; *** *p*<0.001.

## Discussion

This study aimed to examine the gap across the region in Indonesia for PNC utilisation among mothers aged 15–49 years old using the 2017 IDHS data sets. PNC has been the primary strategy to improve maternal health outcomes to reduce the high maternal mortality deaths in Indonesia. Therefore, assessing the regional disparities in PNC may provide evidence for the government to resolve discrepancies in maternal services in Indonesia. This study demonstrated that the prevalence of PNC service utilisation among mothers aged 15–49 years was 70.94%. This finding was higher than other research in Sub-Saharan Africa and Ethiopia with Abebo & Tesfaye (2018) and Tessema *et al.* (2020), respectively finding that 47.9% and 52.48% of women had used PNC service. An intertwined complex factor, such as the health system, maternal health policies, and socio-cultural variations across countries, may hinder women’s use of the PNC service.

Among the geographic groups analysed in this study, mothers who settled in the Central of Indonesia had increased odds of using PNC service, while those who lived in the Eastern of Indonesia had decreased odds. Similarly in Ethiopia ( Sisay *et al.*, 2019), the geographic factor is correlated to the utilisation of PNC service due to the region's level of development and location. Evidence in Indonesia shows that the socio-economic development, such as industrial, housing, public transportation, road facilities and health facilities in the Eastern Indonesia, lagged compared to the Western of Indonesia ( Ministry of Health Republic of Indonesia, 2018; Soewondo *et al.*, 2019; Suparmi *et al.*, 2018). Therefore, it is a call of action to provide equality in developing human resources and infrastructure to reduce the possibility of gaps.

Additionally, mothers who live in Java are 1.46 times more likely to use PNC services than mothers who live in Sulawesi where they are 0.53 less likely to utilise PNC services. Java has dominance development compared to other islands because this island is the centre of the Indonesian government ( Laksono *et al.*, 2020). The Java island oriented and centred development model has harmed maternal health outcomes in Indonesia ( Bappenas, 2018). Natural resources, human resources and facilities must be equal throughout Indonesia, so the gap between islands could be minimised.

This study revealed that the wealth index was significantly linked to PNC service utilisation among mothers aged 15–49 years old in Indonesia. Mothers from the middle households based on the wealth index had PNC service’s utilisation increased by 1.25 greater odds (OR = 1.25; 95% CI = 1.07-1.47) more than the richer mothers (OR = 1.23; 95% CI = 1.04-1.45). However, earlier research is done in Pakistan, Ethiopia, and Tanzania, revealed where mothers from the richer wealth quintile were significantly associated with the utilisation of PNC services ( Berhe *et al.*, 2019; Mohan *et al.*, 2017; Yunus *et al.*, 2013). The odds ratio was quite similar between middle and richer households as shown on this study. Additionally, the previous study in other parts of Ethiopia demonstrated that sociodemographic factors, such as income, did not correlate with the use of PNC services ( Angore *et al.*, 2018). Mothers from richer households were more likely to access the PNC services. Ownerships of consumer goods at home, such as motorcycles and cars, may increase the risk at ease transportation, making them have no strain to access the health facility.

The present study showed that access to the health facility was correlated to PNC service in Indonesia. With mothers who thought that access to the health facility was not a problem, they had an odds ratio of 1.29 greater in using PNC service than mothers who considered access to the health facility difficult. Previous research conducted in Malawi has found a significant association between the health facility and the utilisation of PNC services. Other Ethiopia studies have demonstrated that physical accessibility plays an essential variable in health service utilisation ( Kim *et al.*, 2019; Tarekegn *et al.*, 2014). Access to the health facility is related to the costs incurred, which is influenced by having transportation to the health facility, which is considered expensive. Long and shorter distances, better roads, and better public transportation may increase access to the health facility, primarily in Indonesia, with its massive gaps in development across the country ( Bappenas, 2018).

In this study, mothers having children aged three months increased the likelihood to use PNC services about 1.43 times (OR = 1.43; 95% CI = 1.23-1.66) more than mothers with children aged four months (OR = 1.30; 95% CI = 1.12-1.51). A similar study in Nepal showed that PNC service utilisation in the early postnatal period was most likely due to the motherhood transition period ( Sanjel *et al.*, 2019). The possible reason could be that mothers with fewer children, and younger children, may want information and be petrified of complications during the postnatal period. In the Indonesia setting, the first neonatal examination (KN1) is carried out at 6–48 hours after the baby is born, which is at the same time for the first PNC visit (KF1). The second neonatal examination (KN2) is carried out between 3–7 days with the second PNC visit (KF2). The third neonatal examination (KN3) occurs alongside the third PNC visit (KF3), which is between 8–28 days after birth ( Ministry of Health Republic of Indonesia, 2018).

Also, mothers who have second child had a 0.88 times likelihood to utilize PNC service (OR = 0.88; 95% CI = 0.78-0.99) than mothers having a third child (OR = 0.86; 95% CI = 0.74-0.99). Some studies in Ethiopia and India showed that the higher the child's birth order, the lower the utilisation of PNC services ( Ali & Chauhan, 2020; Sisay *et al.*, 2019). A possible reason could be that mothers who had more children were more likely experienced in motherhood and had appropriate knowledge from previous maternal experiences and childcare, hence restraining PNC service.

This study revealed that mothers who hold higher education qualifications have 2.11 times the chance of utilising PNC visits (OR = 2.11; 95% CI = 1.24-3.59) compared to those with lower-level education (OR = 1.80; 95% CI = 1.09-2.98). Earlier research in Ethiopia showed that a mother's education was significant to PNC utilisation ( Tarekegn *et al.*, 2014). The possible reason could be that educated women have a greater opportunity to be informed and are more aware of seeking advice and treatment from skilled healthcare personnel than uneducated women.

This study demonstrated that the spouse's age was significantly linked to the utilisation of PNC among mothers aged 15–49 years in Indonesia. Mothers who have spouses aged 41–50 have a higher chance of utilising PNC visits than mothers who have spouses aged 31–40. Similarly, in India showed that the spouse's age was associated with PNC's wives' service ( Jungari & Paswan, 2019). Spouse's autonomy and power in decision-making regarding wive's needs, including their health care needs, remained persistent. The spouse's age may be linked to the level of maturity and primary controller, which exacerbates their wives' access to the health service ( Jungari & Paswan, 2019; Sekine & Carter, 2019).

To the best of our knowledge, this is the first study to examine the gaps across the region in utilisation PNC service among mothers aged 15–49 years old in Indonesia, which is one of a country in Southeast Asia that contribute to the global burden of maternal mortality rates in the world. The strengths of our study that we utilised the national and provincial data representatives, which internationally standardised. However, we also note some limitations. The IDHS data analysed in this study was collected using the cross-sectional method and mother’s recall preceding survey prone to the possibility of bias information.

## Conclusion

This study reveals the gap across the region for PNC utilisation among mothers aged 15–49 years old in Indonesia. This study's findings provide evidence to complete the bigger picture for the government to resolve discrepancies in maternal services in Indonesia. The results suggest the need for national policy focuses on service equality, accessible, and reliable implementation to improve postnatal care utilisation among mothers to achieve the maximum results for the Indonesian Universal Health Coverage plan. Future research should explore the interregional gaps and factors that cause maternal health service utilisation by using a different platform.

## Data availability

Data used in this study is available online from the Indonesian 2017 Demographic and Health Survey (DHS) website under the DHS VII recode column. Access to the dataset requires registration and is granted only for legitimate research purposes. A guide for how to apply for dataset access is available at: https://dhsprogram.com/data/Access-Instructions.cfm. Other researchers will be able to access the data set in the same way as the authors and the authors do not have special access rights that others do not have.
