# Supplementary material for: Regional disparities in postnatal care among mothers aged 15-49 years old: An analysis of the Indonesian Demographic and Health Survey 2017
Source: F1000Res. 2021 Aug 16;10:153. Originally published 2021 Feb 26. [Version 2] doi: 10.12688/f1000research.50938.2 (PMC8323067; doi:10.12688/f1000research.50938.2)
Supplement: Peer review history

# Peer review: Reviewer response for version 1

**DOI:** 10.5256/f1000research.54033.r89395

**Published:** 2021-07-29

**Authors:** Kusrini S. Kadar

**Affiliations:** 1 Faculty of Nursing, Universitas Hasanuddin, Makassar, Indonesia; 2 School of Nursing and Midwifery, La Trobe University, Melbourne, Vic, Australia

This is a very interesting study providing information of the utilization of PNC services in Indonesia. The authors have described the gaps across the region clearly. We can see which regions in Indonesia utilized more PNC service as well as other factors contributing to this utilization. 

 As this study using secondary data from Indonesian DHS in 2017, this situation might have changed now, perhaps it is good to add in the title the year of the survey. 

 In the method section, it is not very clear whether the authors explain the method used by the IDHS or the method for this study. The information provided in this section hinders the possibility of others to replicate this study. Need more detailed information, step-by-step of what the authors did in this method section ^1^.

 In the variable, for example, the authors categorized the mothers' age into six groups however there is no reference of this categorizing whether using WHO definition or else. Need to provide more information regarding this section.

 Statistical analysis is already clear and can be replicated. The description of the result also has been clear.

 In the discussion part, the authors' explanation is very repetitive. Every paragraph has a similar pattern where it started from the finding, comparing with previous study and additional explanation. Although this pattern can be accepted, however somehow make this part very boring. The authors tend to start the statement with: *this study inline with.... the result congruent with...consistent with previous study... *If it's possible to change the pattern in every paragraph not only the word that is used.

 Lastly, there are some words that can be changed such as **husband **with **spouse.**

****

 Term for explaining regions in Indonesia can be changed (follow the time zone of Indonesia): 

1. "West of Indonesia" with Western part/regions of Indonesia/Western Indonesia.
2. "Middle of Indonesia" with Central part/regions of Indonesia/Central Indonesia.
3. "East of Indonesia" with Eastern part/regions of Indonesia/Eastern Indonesia.

For language clarity, I encourage the authors to use an English language editing service to produce more clear sentences and paragraphs.

Is the work clearly and accurately presented and does it cite the current literature?

Yes

If applicable, is the statistical analysis and its interpretation appropriate?

I cannot comment. A qualified statistician is required.

Are all the source data underlying the results available to ensure full reproducibility?

Yes

Is the study design appropriate and is the work technically sound?

Partly

Are the conclusions drawn adequately supported by the results?

Yes

Are sufficient details of methods and analysis provided to allow replication by others?

Partly

Reviewer Expertise:

Community health; health education; health promotion; nursing workforce

I confirm that I have read this submission and believe that I have an appropriate level of expertise to confirm that it is of an acceptable scientific standard.

## References

[1] : Predicting Obesity in Adults Using Machine Learning Techniques: An Analysis of Indonesian Basic Health Research 2018.*Front Nutr*.2021;8: 10.3389/fnut.2021.66915566915510.3389/fnut.2021.66915534235168PMC8255629

# Response

**Published:** 2021-08-08

**Authors:** Ferry Efendi

**Affiliations:** Universitas Airlangga, Indonesia

1. We appreciate the reviewer’s positive feedback and recommendations. We have added in the title the year of the survey as suggested. It would now read “Regional disparities in postnatal care among mothers aged 15-49 years old: An analysis of the Indonesian Demographic and Health Survey 2017”.

 2. Thank you for your feedback. The sentence have been revised as follow: This study was a secondary data analysis using the most recent data from the 2017 Indonesian Demographic Data Survey (IDHS) collected by the Inner City Fund (ICF). This study is part of the International Demographic and Health Survey (DHS) program. We clarify that in the data availability section the information required for other researchers to replicate this study. Data used in this study is available online from the Indonesian 2017 Demographic and Health Survey (DHS) website under the DHS VII recode column. Access to the dataset requires registration and is granted only for legitimate research purposes. A guide for how to apply for dataset access is available at: https://dhsprogram.com/data/Access-Instructions.cfm. While the guide for using datasets for analysis is available at https://dhsprogram.com/data/Using-DataSets-for-Analysis.cfm. Other researchers will be able to access the data set in the same way as the authors and the authors do not have special access rights that others do not have. Further, we clarify that unlike other secondary data, DHS secondary data is structured and well organized to produce high-quality data. We have added more information on methods section for the purpose of replicability. 

 Authors categorized the mothers' age into six groups based on the reference from DHS report, we have added the information on the methods section.

 3. We appreciate the reviewer’s positive feedback and recommendations. The discussion has been revised according to the reviewer suggestion. The sentence have been revised as follows:

*for example:*

- Among the geographic groups analyzed in this study, mothers who settled in the Middle of Indonesia had increased odds of using PNC service, while those who lived in the East of Indonesia had decreased odds. Similarly, in Ethiopia.

*Other paragraphs:*

- However, earlier research is done in Pakistan, Ethiopia, and Tanzania, revealed where mothers from the richer wealth quintile were significantly associated with the utilization of PNC services…
- Other Ethiopia studies have demonstrated that physical accessibility plays an essential variable in health service utilization…
- In this study, mothers having children aged three months increased the likelihood to use PNC services about 1.43 times (OR = 1.43; 95% CI = 1.23-1.66) more than mothers with children aged four months (OR = 1.30; 95% CI = 1.12-1.51). A similar study in Nepal showed that PNC service utilization in the early postnatal period was most likely due to the motherhood transition period.

4. We appreciate the reviewer drawing our attention to check words, which has been corrected in our resubmission with “spouse” and term for explaining regions in Indonesia as suggested (Western Indonesia, Central Indonesia and Eastern Indonesia).

 5. The professional English language editing service has been utilized to clarify sentences.

# Peer review: Reviewer response for version 1

**DOI:** 10.5256/f1000research.54033.r80365

**Published:** 2021-03-16

**Authors:** Asmaa Salah Eldin Mohamed Saleh

**Affiliations:** 1 Community Health Nursing, Beni Suef University, Cairo, Egypt

Hello, best greeting - It is my pleasure to review this article, thanks to the editors and authors.

 I found this article was written in good condition by following the scientific rules for writing and I found nothing to be critic on it except just a few notes that do not affect the article structure; like authors mention Java and Jawa in entire the article, I suggest unifying the term mentioned in the article.

 In the conclusion section, the authors mentioned that *“Structured policies are needed to reduce gaps in areas with low service utilization. Developing innovative strategies to address PNC inequality in maternal services to improve maternal health is expected”. *It would be preferred to have clearly applicable recommendations which serve as the solution for the article hypothesis.

 The references in the article must be written in chronologically way in the same pattern for all.

 Table 3: Socio-demographic characteristic of participants PNC in Indonesia based on region (n=13,901). The total sum of (No) use of PNC is not right (4039) it is 4040, you need to review it.

 Many thanks, with my best wishes.

Is the work clearly and accurately presented and does it cite the current literature?

Yes

If applicable, is the statistical analysis and its interpretation appropriate?

Yes

Are all the source data underlying the results available to ensure full reproducibility?

Yes

Is the study design appropriate and is the work technically sound?

Yes

Are the conclusions drawn adequately supported by the results?

Yes

Are sufficient details of methods and analysis provided to allow replication by others?

Yes

Reviewer Expertise:

Community health nursing, maternal and children health nursing, geriatric health nursing, health promotion, health education, occupational health nursing, public health nursing, primary health nursing, rehabilitation nursing.

I confirm that I have read this submission and believe that I have an appropriate level of expertise to confirm that it is of an acceptable scientific standard.

# Response

**Published:** 2021-08-08

**Authors:** Ferry Efendi

**Affiliations:** Universitas Airlangga, Indonesia

1. We thank the reviewer for the thoughtful review of our manuscript. The term mentioned in the article, which is Java, has been complete and corrected.
2. Thank you for your suggestions. We have revised as follows: The results suggest the need for national policy focuses on service equality, accessible and reliable implementation to improve postnatal care utilization among mothers to achieve the maximum results for the Indonesian Universal Health Coverage plan.
3. The reference list has been completed and corrected.
4. We appreciate the reviewer drawing our attention to this error, which has been corrected in our resubmission (4040).
